# Post-acute cardiac complications following SARS-CoV-2 infection in partial lipodystrophy due to *LMNA* gene p.R349W mutation

**DOI:** 10.1007/s40618-022-01795-6

**Published:** 2022-04-06

**Authors:** G. Ceccarini, D. Gilio, S. Magno, C. Pelosini, M. Leverone, C. Miceli, A. Barison, I. Fabiani, M. Emdin, F. Santini

**Affiliations:** 1 Obesity and Lipodystrophy Center, Endocrinology Unit, University Hospital, Pisa, Italy; 2 Chemistry and Endocrinology Laboratory, University Hospital, Pisa, Italy; 3 Cardiology Unit, Ospedale degli Infermi, Biella, Italy; 4 Cardiology Unit, Presidio Ospedaliero di Cittadella, Padova, Italy; 5 Fondazione Toscana Gabriele Monasterio, Pisa, Italy; 6 Institute of Life Sciences, Scuola Superiore Sant’Anna, Pisa, Italy

**Keywords:** Atypical progeroid syndrome, Lipodystrophy, *LMNA* mutation, SARS-CoV-2, COVID-19, Post-acute cardiac complications

## Abstract

**Purpose:**

SARS-CoV-2 infection may cause varying degrees of cardiac injury and the presence of underlying cardiovascular morbidities contributes to the frequency and severity of occurrence of this complication.

Lipodystrophy syndromes are frequently characterized by severe metabolic derangements that represent relevant cardiovascular risk factors. Besides causing lipodystrophy, mutations in the lamin A/C (*LMNA*) gene can lead to a wide spectrum of tissue-specific disorders including cardiac involvement.

**Methods and results:**

We herein examine the case of two patients affected by atypical progeroid syndrome and partial lipodystrophy due to a heterozygous missense *LMNA* mutation c.1045 C > T (p.R349W) who presented initially with mild COVID-19 and developed severe cardiovascular complications within few weeks of SARS-CoV-2 infection. Before being infected with SARS-CoV-2, our patients had cardiovascular morbidities (mild mitral regurgitation in one patient, ischemic heart disease with bifascicular block in the other patient) in adjunct to cardiovascular risk factors, but the SARS-CoV-2 infection contributed to quickly and significantly decompensate their balance.

**Conclusion:**

These findings warn that patients affected by *LMNA* p.R349W mutation and likely other *LMNA* mutations associated with cardiovascular morbidity should be considered at extremely elevated risk of post-acute cardiological manifestations and should therefore undergo a vigilant follow-up after SARS-CoV-2 infection. Both patients developed COVID-19 before the specific vaccination was available to them and this unfortunate situation should remark the importance of vaccination coverage against SARS-CoV-2 infection for all patients affected by lipodystrophy, especially those with underlying comorbidities.

## Introduction

SARS-CoV-2 infection is characterized by respiratory involvement that, in severe cases, can progress to acute respiratory distress syndrome. However, patients infected by SARS-COV-2 may also develop cardiovascular complications that have an adverse impact on prognosis. Several cardiovascular manifestations of COVID-19 disease have been reported [1, 2]. On the other hand, patients with underlying cardiovascular disease and/or cardiovascular risk factors seem to be at a higher threat of SARS-CoV-2 infection and of developing cardiac complications [1].

We herein describe the clinical history of two patients affected by atypical progeroid syndrome (APS) and partial lipodystrophy caused by a heterozygous missense lamin A/C gene (*LMNA*) mutation c.1045 C > T (p.R349W), who experienced a clinically significant worsening of their underlying cardiovascular diseases following SARS-CoV-2 infection.

APS is caused by different *LMNA* mutations that have in common an early and accelerated aging process and is also characterized by variable degrees of fat loss (partial or generalized lipodystrophy), metabolic alterations and comorbidities that affect skeleton, muscles and heart. Twenty patients with *LMNA* p.R349W mutation have been reported so far, all presenting with very similar features [3, 4]. Lipodystrophic patients display reduced or absent adipose tissue amounts and this condition cause a proportional reduction of circulating levels of adipocyte-derived hormones, especially leptin. On this note, it is worth mentioning that leptin deficient patients are considered at increased mortality risk for infectious diseases [5] and therefore more serious COVID-19 manifestations in lipodystrophic patients who develop SARS-CoV-2 infection (which among other endocrine organs interestingly target the adipose tissue [6]) may be expected. However, the lack of scientific reports in this regard, does not allow confirming this hypothesis.

## Results and case description

### Case 1

Patient 1 is a 46-year-old Caucasian female affected by APS due to *LMNA* Gene p.R349W mutation [4]. She had precocious aging traits such as short stature, mandibular hypoplasia, beaked nose, and partial alopecia, partial lipodystrophy, sensorineural hearing impairment, proteinuric nephropathy, hypertension and metabolic abnormalities including dyslipidemia, hepatic steatosis, insulin resistance, and impaired glucose tolerance. She also had a concomitant diagnosis of monoclonal gammopathy of undetermined significance (MGUS). Her father died suddenly at the age of 37 years. Her last cardiological evaluation, carried out in March 2020, revealed mild left ventricular hypertrophy, normal systolic function (ejection fraction 56%), and mild mitral and tricuspid regurgitation; 24 h holter electrocardiography showed paroxysmal supraventricular tachycardia which was treated with beta-blockers, while high-sensitivity troponin T resulted within normal limits (hs-TnT 5 ng/L). In October 2018, a cardiac magnetic resonance imaging (MRI) confirmed a normal biventricular function, with no significant tissue abnormalities.

At the end of October 2020, during the second wave of COVID-19, 7 months after her last cardiological evaluation, she became infected with SARS-CoV-2 with mild symptoms including anosmia, ageusia, asthenia, fatigue and dyspnea which did not require specific pharmacological treatments allowing her to continue the chronic therapies. The patient’s 18-year-old son, also affected by atypical progeroid syndrome, was infected by SARS-CoV-2 at the same time and complained of fever, cold, anosmia and ageusia. He has the same key features of the mother, but does not display yet metabolic alterations and cardiovascular disease [4].

On January 1st 2021, the patient presented with epigastric pain and palpitations followed by the onset of peripheral edema. She was admitted for heart failure associated with high rate atrial fibrillation (140 beats/min). Echocardiography showed severe left ventricular systolic dysfunction with diffuse hypokinesia and severe mitral regurgitation. The N-terminal prohormone of brain natriuretic peptide (NT-proBNP) levels were elevated (6097 pg/mL). Biochemical analysis, under statin and metformin treatment showed normal glycated hemoglobin, triglycerides, total cholesterol and fractions, liver and renal function. Creatinine-kinase and lactate dehydrogenase (LDH) levels were also normal. During hospitalization, diuretic therapy was initiated together with high-dose beta-blocker (metoprolol) and anticoagulation (warfarin); diltiazem was then added to metoprolol despite her known LV dysfunction, for better rate control. After heart rate normalization, transthoracic echocardiography revealed partial recovery of LV systolic function (LVEF of 45%) and moderate mitral regurgitation. The patient underwent coronary angiography, which detected atherosclerotic plaques (proximal and middle regions of right coronary artery-RCA), not hemodynamically significant. In May 2021, 4 months after discharge, the patient was in fairly good hemodynamic compensation. She was still on diuretic therapy and a combination of high-dose metoprolol and diltiazem because of persistent atrial fibrillation with a relatively high ventricular response (90 beats/min); her echocardiography showed normal ventricular volumes and systolic function (LVEF = 58%) with moderate mitral regurgitation.

**Fig. 1:**
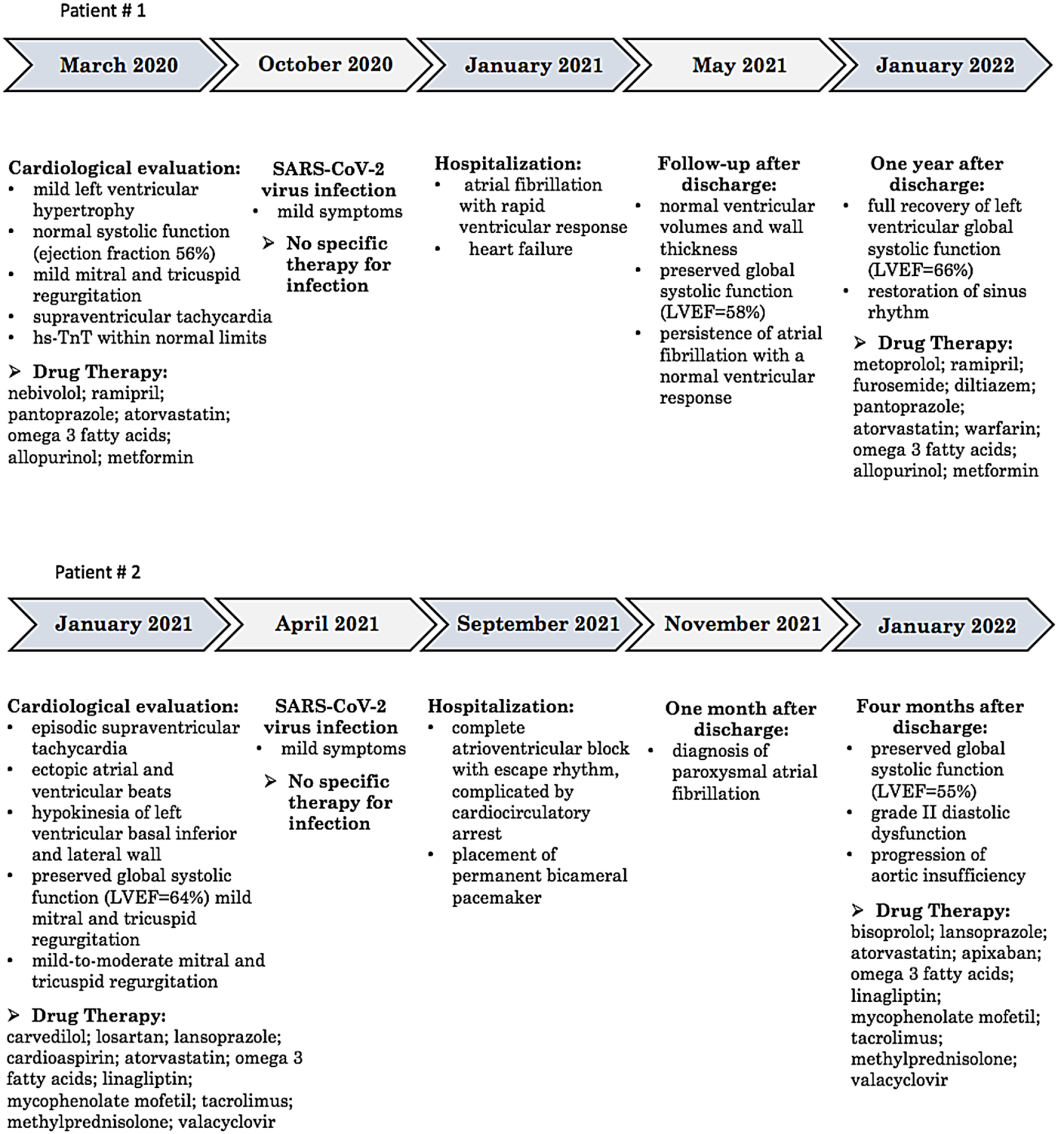
Timeline of key clinical events and drug treatments before and after SARS-CoV-2 infection in the two patients affected by atypical progeroid syndrome and partial lipodystrophy due to a heterozygous missense lamin A/C gene (*LMNA*) mutation c.1045 C > T (p.R349W)

Last cardiological evaluation, carried out in March 2022 under the same therapy, revealed a full recovery of left ventricular global systolic function (LVEF = 66%) and the restoration of sinus rhythm (heart rate 90 beats/min). The pharmacological treatments before and after the cardiac complications are summarized in Fig. 1.

Worth mentioning that the patient’s son did not develop complications after SARS-CoV-2 infection.

### Case 2

Patient 2 is a 41-year-old Caucasian male affected by APS due to *LMNA* Gene p.R349W mutation [4]. At physical exam, he presents with short stature, beaked nose, partial alopecia, skin atrophy, thin lips and small mandible associated with partial lipodystrophy, reduced sensorineural hearing acuity, diabetes mellitus type 2, dyslipidemia, liver steatosis and hypertension. At the age of 27, he was diagnosed with a neuroendocrine (NET) tumor which was treated with hemicolectomy followed by liver transplantation because of liver metastases and at the age of 34, he had a kidney transplantation for kidney failure due to focal segmental glomerulosclerosis.

In January 2021, his ECG showed sinus rhythm with bifascicular block (complete right bundle branch block and left anterior hemiblock); a 24 h holter monitoring showed episodes of supraventricular tachycardia, and ectopic atrial and ventricular beats, with no apparent brady-arrhythmias; for this reason, he was treated with beta-blockers. Echocardiography revealed mild-to-moderate mitral and tricuspid regurgitation; hypokinesia of left ventricular basal inferior and lateral wall with preserved global systolic function (LVEF = 64%). The patient had undergone a coronary angiography just before kidney transplant which detected atherosclerotic plaques, not hemodynamically significant. He had also undergone a cardiac MRI scan in 2020, showing signs of ischemic fibrosis in the left anterior descending and circumflex territories.

In April 2021, the patient was infected by SARS-CoV-2 with very mild symptoms complaining only of anosmia and ageusia which did not require specific pharmacological treatments allowing him to continue the chronic therapies.

On the 30th of September 2021, he presented with a syncopal episode associated with vomiting. At the emergency room electrocardiography showed complete atrioventricular block with escape rhythm (heart rate 35 beats/min). Echocardiography revealed left ventricular hypertrophy and basal inferior hypokinesis with preserved global systolic function (LVEF = 58%), the patient had cardiocirculatory arrest due to asystole which required cardiopulmonary resuscitation, atropine and adrenalin with subsequent restoration of sinus rhythm without neurological deficit. Once the patient was hemodynamically stabilized, he was fitted with a permanent bicameral pacemaker. During hospitalization, a CT scan revealed diffuse consolidation in the lower lobe of both lungs and bilateral pleural effusion. For this reason, he was treated with antibiotics and mycophenolate transiently withdrawn. During hospitalization, glucose and lipid profiles did not show any significant alteration (under statin and linagliptin treatment); liver and renal function were also normal. At discharge, electrocardiography showed pacemaker driven rhythm with normal heart rate (75 beats/min) and the lung consolidations were no longer detected on CT scan.

In November 2021, 1 month after discharge, the patient was diagnosed with paroxysmal atrial fibrillation and anticoagulant therapy was initiated.

The last cardiological evaluation, carried out in January 2022, showed preserved global systolic function (LVEF = 55%), grade II diastolic dysfunction and moderate mitral regurgitation (significantly worsened compared to January 2021). The pharmacological treatments before and after the cardiac complications are summarized in Fig. 1.

## Discussion

Patients with underlying cardiovascular disease and/or cardiovascular risk factors are at higher risk of developing severe COVID-19 [7–10]. Moreover, cardiovascular disorders, hypertension and diabetes are among the most important negative prognostic factors for COVID-19 [11, 12]. Lipodystrophic syndromes are rare disorders characterized by subcutaneous fat loss, in the absence of a nutritional deprivation or a catabolic state [13, 14]. Lipodystrophy syndromes are frequently characterized by severe metabolic derangements which represent relevant cardiovascular risk factors [15]. Additionally, these patients display reduced levels of the adipocyte-derived hormone leptin, a modulator of proliferation and function of T lymphocytes, phagocytosis by macrophages, neutrophil chemotaxis and bone marrow function [16–18]. Thus, as leptin deficient patients are considered more susceptible to infectious diseases [5], more serious consequences after SARS-CoV-2 infection would in theory be expected in them. A high prevalence of SARS-CoV-2 infection in congenital generalized lipodystrophic Brazilian patients, with a good outcome in all of them, was recently reported, but the mean age of the infected patients was very young, thus making difficult to extend these findings to the entire population of affected patients [19]. At this time, no other report in this regard is present in the literature.

Mutations in the *LMNA* gene, besides causing lipodystrophy, lead to a wide spectrum of tissue-specific disorders. We have previously reported a high prevalence of cardiac morbidities among patients with p.R349W *LMNA* mutation: more than 80% of patients display rhythm disorders, 62% of them cardiac valvular abnormalities including mitral, aortic, or tricuspid regurgitation, and 36% cardiomyopathy [4].

SARS-CoV-2 infection may cause varying degrees of cardiac injury and the presence of underlying cardiovascular morbidities contributes to the frequency and severity of occurrence of this complication. In a study on 113 deceased COVID-19 patients, common complications included acute cardiac injury (77%) and signs of heart failure (49%) [20]. One of the possible pathophysiological mechanisms is the direct myocardial injury: SARS-CoV-2 enters human cells by binding to angiotensin converting enzyme 2 (ACE-2) leading to acute injury in tissues where it is expressed [21]. ACE-2 is widely expressed in the human body, including endocrine tissues; for this reason, endocrine complications, in COVID-19 patients, should be investigated, especially in case of deterioration of the clinical status [22, 23]. It has also been hypothesized that cytokine storm, high catecholamine levels and profound hypoxia may contribute to cardiomyocyte damage and acutely reduce cardiac function. Atrial fibrillation or other tachy-arrhythmias triggered by systemic inflammation, hypoxemia, metabolic derangements or myocarditis are other possible precipitants of heart failure [24]. Even though cardiovascular manifestations maybe the initial presentation of COVID-19 or may appear throughout the course of infection, emerging reports suggest that the cardiovascular signs and symptoms may persist beyond the acute setting [25, 26]. It has been reported that patients are at a higher risk of death and may exhibit hypertension, cardiac dysrhythmias, chest pain, ischemic and non-ischemic heart disease, pericarditis, myocarditis, heart failure and thromboembolic disorders beyond the first 30 days of COVID-19 [25, 26]. This risk is evident even among individuals who were not hospitalized during the acute phase of SARS-CoV-2 infection and in young survivors (age ≤ 65 years) of acute COVID-19 [24]. Beyond the first 30 days of COVID-19, there is also evidence of an increased prescription of beta-blockers, calcium channel blockers, loop diuretic agents, thiazide diuretic agents, and antiarrhythmic drugs [25]. The post-acute cardiovascular sequelae of infection seem to be substantial also at 12 months [25]. In a study on 97 patients, who had been hospitalized with non-severe COVID-19 and who did not present overt cardiac manifestations during the SARS-CoV-2 infection, a systematic cardiac screening for latent cardiac abnormalities was performed up to four weeks after hospital discharge. Cardiac abnormalities were detected in 42.3% of them including sinus bradycardia (29.9%), newly detected T-wave abnormality (8.2%), elevated troponin level (6.2%), newly detected atrial fibrillation (1.0%), and newly detected left ventricular systolic dysfunction with elevated NT-proBNP level (1.0%) [27]. In a multivariate analysis, only age remained associated with newly detected cardiac abnormalities among them [27]. It has been reported that 14% of deceased COVID 19 patients had lymphocytic myocarditis [28], it is therefore reasonable to think that the observed late troponin elevation [27] may reflect a prolonged cytopathogenic effect of the virus on cardiomyocytes. However, it is fair to say that the disease mechanisms involved in post-acute manifestations of COVID-19 are not well understood: some of the manifestations may be driven by a late-onset direct effect of the viral infection or alternatively may be explained by the virus persisting in immune-privileged sites or an aberrant immune response, including autoimmunity [29].

Gatto et al. described a case of a non-hospitalized 47 years old man, with no relevant cardiovascular and medical history, who developed a sinus bradycardia 2 months after mild SARS-CoV-2 infection, requiring the implantation of a loop recorder with remote monitoring [30]. It has also been hypothesized that some of these complications may result from a dysfunction of the autonomic nervous system due to a direct viral damage or to an autoimmune mechanism [31, 32]. We herein report the clinical history of two patients affected by APS and partial lipodystrophy due to *LMNA* p.R349W mutation who displayed mild COVID-19 symptoms and were not hospitalized. However, both patients developed severe cardiovascular complications within few weeks after resolution of SARS-CoV-2 infection (Fig. 1).

One patient displayed atrial fibrillation with a rapid ventricular response and heart failure, and the other one had a cardiac arrest which required cardiopulmonary resuscitation and implantation of a permanent dual-chamber pacemaker. Before contracting the SARS-CoV-2 infection, both patients had cardiovascular comorbidities (mild mitral regurgitation in one patient, ischemic heart disease with bifascicular block in the other patient) in adjunct to multiple cardiovascular risk factors such as hypertension, dyslipidemia and diabetes mellitus type 2. Nevertheless, the coincidence in timing with the SARS-CoV-2 infection is highly suspicious for a direct or indirect effect of the viral infection in causing further cardiac damage and plunging an unstable balance. In addition, it is worth emphasizing that the biological age of both patients is older compared to their chronological age due to the progeroid component of their syndrome and aging is clearly an identified risk factor for newly detected cardiac abnormalities among COVID-19 survivors [27]. In accordance with this evidence, the very young son of patient 1 did not report any complication after SARS-CoV-2 infection.

The association between *LMNA* mutations, lipodystrophy and cardiomyopathy has been amply reported in the literature and the prevalence of all cardiac disorders increases with age [33, 34]. These findings are therefore a warning, in our opinion, that patients with *LMNA* p.R349W mutation (and likely many other *LMNA* mutations including those causing familial partial lipodysrophy), should be considered at increased risk of late onset cardiological manifestations of COVID-19 and therefore undergo vigilant follow-up after SARS-CoV-2 infection. In this regard, we suggest to sensitize patients on possible warning signs of cardiac complication such as dyspnea, epigastric pain, palpitations, peripheral edema, fatigue and increase medical monitoring, whenever possible, by an accurate cardiological evaluation (based on assessment of BNP, EKG, echocardiography) at least once within 6 s after infection.

The recent development of the European lipodystrophy registry may unveil the risks related to SARS-CoV-2 infection in lipodystrophic patients [35]. Both patients herein described developed COVID-19 before the specific vaccination was available; this unfortunate circumstance should once more remark the importance of vaccination coverage against SARS-CoV-2 infection for all patients affected by lipodystrophy, especially those with underlying comorbidities.

## Data Availability

All data generated or analyzed during this study are included in this published article or in the data repositories listed in the References.
